# Volume Stability and Frost Resistance of High–Ductility Magnesium Phosphate Cementitious Concrete

**DOI:** 10.3390/ma17112522

**Published:** 2024-05-23

**Authors:** Lijuan Chai, Zhonghua Yue, Zhichun Chen, Gaoyu Fan, Liuye Wang

**Affiliations:** 1 College of Civil Engineering, Taiyuan University of Technology, Taiyuan 030024, China; chailijuan@tyut.edu.cn (L.C.); yuezhonghua567@163.com (Z.Y.); 17856341665@163.com (G.F.); wangly144@163.com (L.W.); 2 School of Materials Science and Engineering, Southeast University, Nanjing 211189, China; 3 Technical Supervision and Research Center of the Building Materials Industry, Beijing 100024, China

**Keywords:** high-ductility magnesium phosphate cementitious concrete (HD–MPCC), deformation property, frost property, tensile property, flexural property, pore structure

## Abstract

To address the issue of pavement cracking due to brittle concrete in road and bridge engineering, this study explores the use of high–ductility magnesium phosphate cementitious concrete (HD–MPCC) for rapid repairs. The deformation and frost properties of HD–MPCC are analyzed to assess its suitability for this application. Deformation properties were tested for HD–MPCC specimens cured in both air and water. Subsequent tests focused on the frost performance and mechanical properties after freeze–thaw cycles. A mercury penetration technique was utilized to examine the pore structure. The findings reveal that the expansion deformation of HD–MPCC increases with curing age in both air and water conditions, and the quantitative relationship between the expansion deformation and curing age of HD–MPCC was analyzed. Additionally, the freeze–thaw cycles led to a decrease in mass loss, the relative dynamic elastic modulus, the ultimate tensile strength, the ultimate tensile strain, the flexural strength, and the peak deflection. The volume fraction of harmless and less harmful pores gradually decreased as the freeze–thaw cycle increased, while the volume fraction of more harmful pores increased, resulting in a decrease in the strength, ultimate tensile strain, and peak deflection.

## 1. Introduction

In China, most road and bridge pavements are constructed using asphalt concrete. Asphalt concrete has poor tensile deformation capacity, leading to damage to the concrete pavements of roads and bridges under vehicle loads. Once the concrete cracks, the cracks deteriorate rapidly, leading to mesh cracking. Considering the stess characteristics and reasons for the damage of concrete pavement, as well as the necessity for rapid traffic reopening, it is imperative to develop cementitious materials with the same or a higher strength grade, high ductility, and rapid hardening for repairing road and bridge pavements.

Magnesium phosphate cement (MPC) is a new type of inorganic cementitious material composed of magnesium oxide, soluble phosphate salts, and retarders in appropriate proportions [1,2]. It has the characteristics of rapid setting and hardening and a low-carbon footprint and is extensively applicable for swift work in engineering projects [3,4,5].

High-ductility concrete (HDC) exhibits multiple crack characteristics under axial tensile loads, with an ultimate tensile strain of no less than 0.5% and an average crack width not exceeding 200 μm after curing for 28 days [6]. Although Portland cement, fly ash, Japanese Kuraray polyvinyl alcohol (PVA) fibers, and fine quartz sand have been used to formulate HDC, the high costs of Japanese Kuraray PVA fibers and fine quartz sand limit their widespread adoption [7,8,9]. Recent studies [10,11,12] have explored the substitution of fine quartz sand with river sand and Japanese Kuraray PVA fibers with domestically produced PVA fibers to create a more cost–effective HDC.

Building on the methodology of low–cost HDC preparation, using MPC as a binder material to prepare high–ductility magnesium phosphate cementitious concrete (HD–MPCC) offers a promising solution for the efficient and rapid repair of cracked concrete pavement in road and bridge engineering. It is essential to examine the volume stability and freeze–thaw resistance of HD–MPCC to confirm its suitability for these applications.

Feng et al. [13,14] have prepared MPC cement using magnesium oxide, potassium dihydrogen phosphate, and borax, combining it with fly ash, silica fume, fine quartz sand, Japanese Kuraray PVA fibers, and water to produce HD–MPCC material. After 7 days of curing, HD–MPCC exhibited a compressive strength ranging from 30 to 45 MPa and an ultimate tensile strain between 0.5%and 4%. Nevertheless, the cost associated with Japanese Kuraray PVA fibers and fine quartz sand make HD–MPCC prohibitively expensive for broad engineering use, and studies on its deformation performance and freeze–thaw resistance remain lacking. 

Qiao et al. [15], Wen et al. [16], and Ma et al. [17] demonstrated that MPC mortar exhibited less drying shrinkage than that of traditional cement mortar. Li et al. [18] noted expansion deformation in specimens containing MPC and fly ash. Jiang et al. [19] observed that the expansion deformation of MPC mortar increased with increasing age. Similarly, Lu et al. [20] found that the expansion deformation of MPC generally increased with increasing age when using a mix of magnesium oxide, ammonium dihydrogen phosphate, borax, fly ash, metakaolin, and water glass. Factors influencing the volume stability of MPC included the M/P ratio, retarder content, and water–to–binder and sand–to–binder ratios [21,22,23]. Previous studies have yielded inconsistent conclusions on whether different MPC mix ratios result in shrinkage or expansion, underscoring the need for further analysis of the deformation performance of various MPC mixtures. Additionally, the literature has not addressed the volume stability of HDC utilizing MPC as a binder.

Yang et al. [24] suggested that using magnesium oxide and ammonium dihydrogen phosphate to prepare MPC resulted in the presence of ammonia gas bubbles within the MPC, which had a similar effect on enhancing freeze–thaw resistance, giving the MPC good freeze–thaw resistance. Ma et al. [17] observed that with an increase in freeze–thaw cycles, the mass loss of MPC mortar increased, leading to a decrease in the remaining compressive strength. Jia et al. [25] found that the mass loss of MPC mortar gradually increased with more freeze–thaw cycles, and the compressive and flexural strength of the MPC mortar initially increased and then decreased. Tao et al. [26] also found that increasing freeze–thaw cycles elevated the mass loss of MPC concrete and reduced its relative dynamic elastic modulus. Ma et al. [27] reported a decrease in the compressive strength of MPC mortar with an increase in freeze–thaw cycles. Wu et al. [28] documented a reduction in both the compressive and flexural strength of MPC mortar with more freeze–thaw cycles. These studies collectively suggest that mortar specimens made with MPC as a binder material experience strength loss after numerous freeze–thaw cycles. In the preparation of HD–MPCC material, the addition of fibers can help resist cracking during freeze–thaw cycles, improving freeze–thaw resistance [12,29]. Thus, the behavior of HD–MPCC post–freeze–thaw cycles warrants additional investigation.

In this study, HD–MPCC material was prepared, and the deformation properties of HD–MPCC cured in air and water were analyzed. In addition, the frost properties and tensile–flexural properties of HD–MPCC after freeze–thaw cycles were evaluated. Additionally, the pore structure of HD–MPCC after different freeze–thaw cycles was characterized. This research not only provides a volume stability analysis of HD–MPCC in the rapid repair engineering field but also explores the feasibility of its use in severely cold regions. 

## 2. Materials and Methods

### 2.1. Raw Materials

According to the code in [30], the MPC was prepared. MPC was used as the binder material (B), prepared through the acid–base neutralization reaction of magnesium oxide (MgO, abbreviated as M) and ammonium dihydrogen phosphate (NH_4_H_2_PO_4_, abbreviated as P). Borax (Na_2_B_4_O_7_·10H_2_O, abbreviated as BO) was adopted as a retarder to slow down the setting time of MPC. Fly ash (FA) of Grade II was utilized. A powder solid polycarboxylate superplasticizer (PS) was used as a water reducer. Lithium (Li) was employed as an early strength agent to enhance the early strength of the material. River sand (RS) was used as fine aggregate with a maximum particle size of 1.18 mm and a fineness modulus of 1.0. Domestically produced PVA fibers were used, and the physical and mechanical properties of the fibers are shown in Table 1. Tap water (W) was used for mixing.

### 2.2. Mix Proportion 

Through the performance optimization design [31], the HD–MPCC mixture was selected, which met the property criteria of a cubic compressive strength of more than 40 MPa, an ultimate tensile strain of more than 1.0%, and an average crack width of less than 200 μm [6,32]. The mixture design of HD–MPCC is shown in Table 2.

### 2.3. Specimen Process

**Figure 1:**
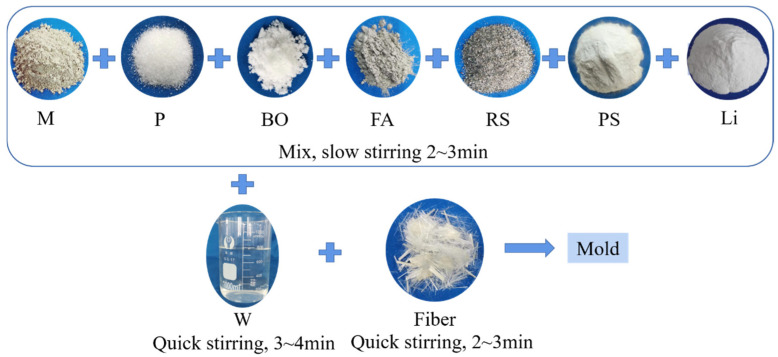
Preparation process for HD–MPCC.

First, we added the M, P, BO, FA, PS, Li, and RS into the mixer and stirred slowly for 2~3 min. Secondly, we added W into the mixer and stirred quickly for 3~4 min. The preparation process for HD–MPCC is shown in Figure 1. After the specimen was cured in air for 6 h, it was demolded, and the macroscopic and microscopic performance of HD–MPCC were tested. For the air conditions, the temperature was (20 ± 2) °C, and the humidity was (30 ± 2)%.

### 2.4. Test Methods

#### 2.4.1. Mechanical Properties

(1) Cubic compressive strength:

The cubic compressive strength test of HD–MPCC followed the specification in [6], with specimen dimensions of 100 mm × 100 mm × 100 mm, and three specimens were used for each group. The cubic compressive strength of HD–MPCC cured for 6h was tested using a 300 T pressure testing machine at a loading rate of 0.6 MPa/s.

**Figure 2:**
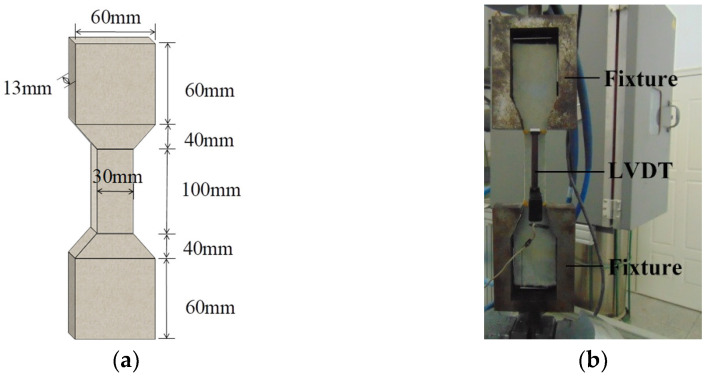
Test device for the tensile property. (**a**) Dimensions of specimen. (**b**) Strain and LVDT device.

(2) Tensile properties:

The tensile performance test of HD–MPCC cured for 6 h was conducted according to the specification in [6]. The specimen dimensions were as shown in Figure 2a, with three specimens used for each group. A displacement gauge (LVDT) was placed in the middle region of the specimen to monitor tensile strain, as shown in Figure 2b. The test was conducted using a tensile testing machine with a displacement loading rate set at 0.002 mm/s.

#### 2.4.2. Deformation Performance

The deformation performance of HD–MPCC cured in air and water for 6 h, 1 d, 3 d, 7 d, 14 d, 28 d, 56 d, 90 d, and 180 d was measured, which can be used to evaluate the volume stability of repair material.

**Figure 3:**
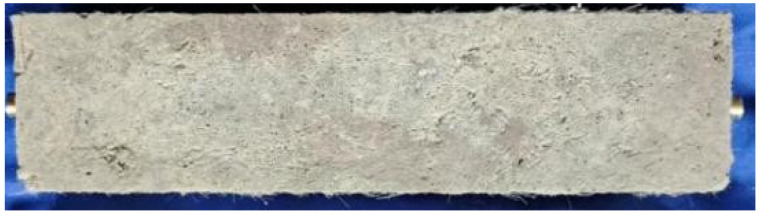
Test specimen for the deformation property.

The deformation performance test method for the HD–MPCC specimens was taken from the standard in [33], with specimen dimensions of 40 mm × 40 mm × 160 mm, and three specimens were tested. Copper shrinkage heads were embedded at both ends of the specimen, with embedding depths of 10 mm on each side, as shown in Figure 3 for the deformation performance of the specimens. The volume stability of HD–MPCC was characterized by testing the linear deformation of HD–MPCC under air curing and water curing conditions. The linear deformation was calculated according to Formula (1).

(1) $$S_{t}=\frac{L_{t}-L_{0}}{140}$$

where *L*_t_ means the measured deformation of the HD–MPCC specimen after curing in water or air for a specified period of time. 

*L*_0_ means the initial length of the HD–MPCC specimen after it has been demolded.

*S*_t_ means the calculated deformation of HD–MPCC after curing for a time t.

The denominator of 140 means the effective length, which was calculated as the specimen length minus the two contraction heads’ lengths buried inside the specimen. The unit was mm in the formula. 

#### 2.4.3. Frost Resistance

According to the method presented in [34], the frost resistance property of HD–MPCC was analyzed. A prism specimen with dimensions of 100 mm × 100 mm × 400 mm was used to conduct the freeze–thaw cycle, and the amount was 3 for every group. Based on the rapid freezing method, the appearance, mass loss, and relative dynamic elastic modulus of HD–MPCC after experiencing freeze–thaw cycles ranging from 0 to 300 were tested. 

The tensile property and flexural property Of HD–MPCC after suffering 0~300 freeze–thaw cycles were measured. The test device for measuring the tensile property of HD–MPCC is shown in Figure 2a, and the amount was 3 for every group. A specimen with dimensions of 100 mm × 100 mm × 400 mm was adopted to assess the flexural property of HD–MPCC, and the amount was 3 for every group. 

#### 2.4.4. Pore Structure

The mercury penetration test was used to evaluate the pore structure of HD–MPCC pure paste after 0 to 300 freeze–thaw cycles, which can reveal the macroscopic property mechanism. 

## 3. Results and Discussion

### 3.1. Mechanical Performance

(1) Cubic compressive strength:

The cubic compressive strength of the HD–MPCC is 41.9 MPa, meeting the strength requirement.

**Figure 4:**
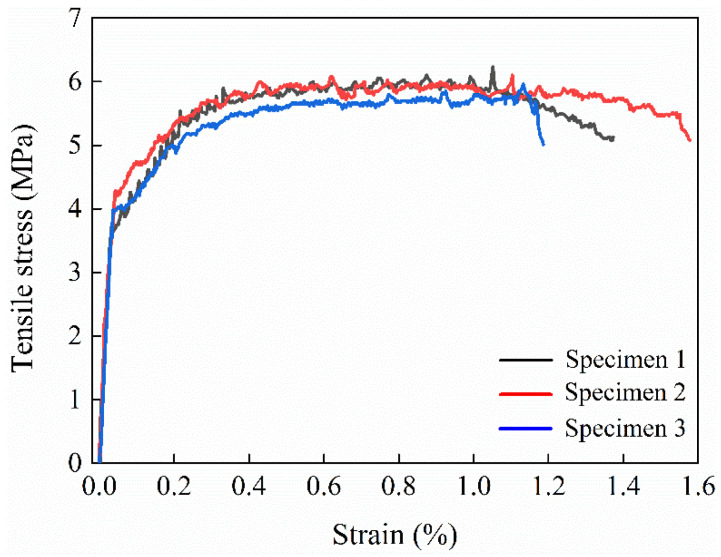
Tensile stress–strain relationship in HD–MPCC [31].

(2) Tensile properties:

The ultimate tensile strength of the HD–MPCC is 6.1 MPa, the ultimate tensile strain is 1.10%, and the average crack width is 117 μm, all meeting the criteria for high ductility. The tensile stress–strain relationship in the HD–MPCC is presented in Figure 4.

The tensile stress–strain relationship in the HD–MPCC exhibits four stages, namely the linear, nonlinear, strain hardening, and strain softening stages. 

Initially, during the early loading stage, the matrix bears the stress, and the relationship between stress and strain is shown to be linear. 

As the loads increase, internal damages in the matrix escalate, resulting in nonlinear stress–strain behavior. 

As the load exceeds the matrix strength, the first crack emerges, accompanied by the first drop point. Despite the decreasing stress, the fiber bridging effect transfers the stress to the surrounding uncracked matrix, leading to increased stress within the specimen. The curve shows the “drop–up–drop…” phenomenon due to the repetitive action of matrix crack, fiber bridges, matrix crack, etc., demonstrating a strain hardening characteristic. 

When the applied load reaches the bearing capacity of the HD–MPCC specimen, the crack widens, accompanied by a decrease in load and an increase in strain, reflecting strain softening behavior in the curve.

### 3.2. Relationship between Age and Deformation in HD–MPCC

#### 3.2.1. Deformation Performance

**Figure 5:**
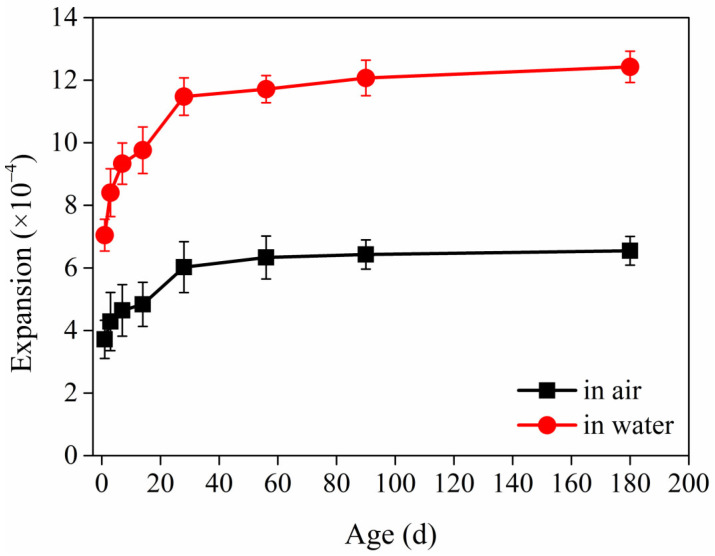
Expansion deformation of HD–MPCC cured in air and water.

The deformation properties of the HD–MPCC during curing in air and water for durations of 1 day to 180 days are shown in Figure 5. As the curing age increases, the expansion deformation of the HD–MPCC increases. Within the initial curing period of 1 day to 28 days, the increasing trend of expansion deformation for the HD–MPCC is obvious. However, beyond 28 days of curing, this increasing trend of expansion deformation becomes less evident. Comparatively, for specimens cured for the same duration, those in water exhibit greater expansion deformation than those in air.

There is a combination of expansion and shrinkage for the HD–MPCC. When the HD–MPCC is cured in air, the water in the capillary will reduce, and the capillary shrinks, resulting in a shrinkage of the HD–MPCC. However, the main hydration product, such as crystallization struvite, has a larger volume [35]. The hydration of magnesium oxide to form magnesium hydroxide can also cause volume expansion. In addition, the PVA fiber can restrict deformation. Therefore, the HD–MPCC shows expansion deformation considering comprehensive factors. 

In the early stages of curing, the HD–MPCC exhibits rapid expansion deformation due to its fast hydration rate and abundant struvite content. As age increases, the hydration products of the HD–MPCC can hinder the hydration process and slow down the rate of hydration. After a curing period exceeding 28 days, the hydration process inside the HD–MPCC stabilizes, leading to a gradual reduction in the linear expansion rate of the specimens.

As the HD–MPCC cures in water, a portion of the water participates in the hydration reaction to generate hydration products, while another portion of the water enters the internal pores of the specimens, causing expansion. Additionally, magnesium oxide hydrolyzes to form magnesium hydroxide as the HD–MPCC cures in water, further contributing to the increased expansion deformation of the specimens. Although the hydration degree of the MPC will increase in water, the struvite will decompose in the water. The HD–MPCC shows expansion deformation due to multiple factors. The capillary will not shrink when the HD–MPCC is cured in water. The expansion deformation of the HD–MPCC specimen under water curing is greater than that of specimens cured in air.

#### 3.2.2. Relationship between Deformation and Time Cured in Air in HD–MPCC

With reference to the shrinkage of concrete, as listed in Formulas (2)~(5), a quantitative relationship between the deformation of the HD–MPCC and curing age is derived.

(2) $$S(t)=\frac{t}{a+bt}\;\;\;t\geq 1$$

(3) S(t)=a+bln(t+1)   t≥1

(4) $$S(t)=S{\left(t\right)}_{,\infty }(1-ae^{bt})\;\;\;t\geq 1$$

(5) $$S(t)=S{\left(t\right)}_{,\infty }+Ae^{at}+Be^{bt}\;\;\;t\geq 1$$

where *a*, *b*, *A*, and *B*—experimental constants;

*S*(t), *_∞_*—final deformation value, unit: ×10^−4^;

*S*(t)—deformation value of specimen at a curing age of t, unit: ×10^−4^;

*t*—curing age, unit: d.

The relationship between the deformation of the HD–MPCC cured in air and age is fitted through Formulas (2)~(5), and the fitting correlation degree is highest when using Formula (5). Fitting Formula (6) is obtained, and the fitting correlation degree is found to be 0.97.

(6) $$S(t)=6.55-2.76e^{-0.052t}-0.135e^{-0.01t}\;\;\;t\geq 1$$

**Figure 6:**
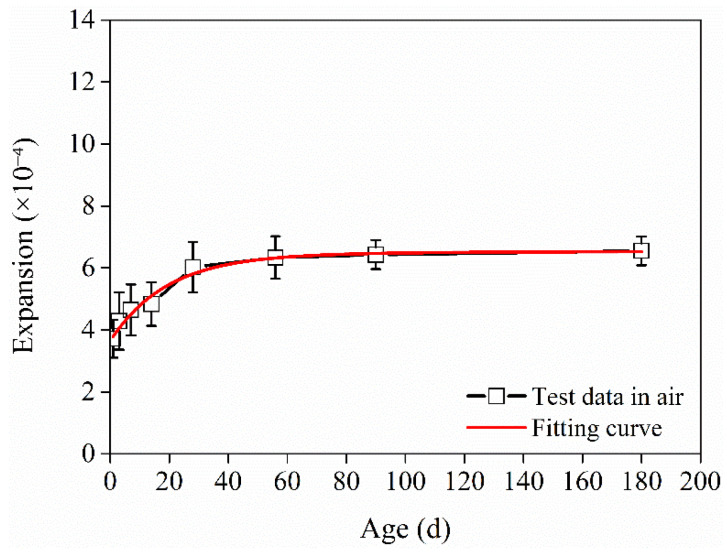
Test data and fitting curve of relationship between deformation and curing age for HD–MPCC cured in air.

The test and fit relationship curves between the expansion deformation of the HD–MPCC cured in air and age are shown in Figure 6.

#### 3.2.3. Relationship between Deformation and Time Cured in Water in HD–MPCC

The relationship between the deformation of the HD–MPCC cured in water and age is fitted through Formulas (2)~(5), and the fitting correlation degree is highest when using Formula (5). Fitting Formula (7) is obtained, and the fitting correlation degree is found to be 0.98.

(7) $$S(t)=12.43-3.566e^{-0.147t}-2.242e^{-0.022t}\;\;\;t\geq 1$$

**Figure 7:**
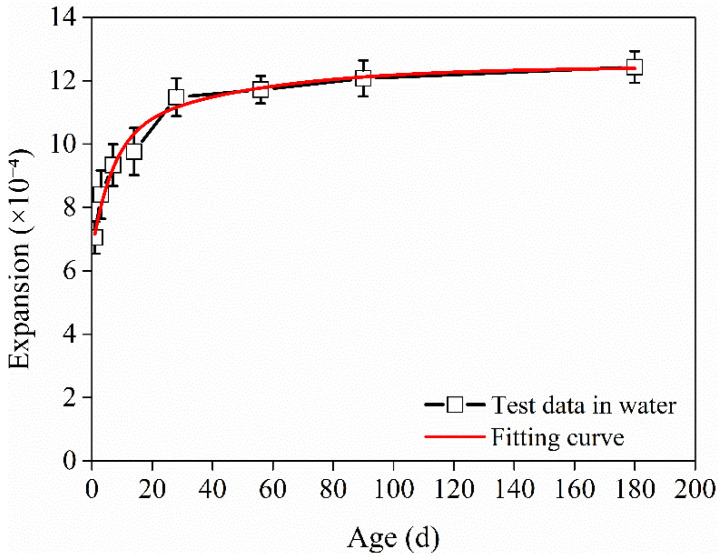
Test data and fitting curve of relationship between deformation and curing age for HD–MPCC cured in water.

The test and fit relationship curves between the expansion deformation of the HD–MPCC in water and age are shown in Figure 7.

### 3.3. Frost Resistance

#### 3.3.1. Appearance of Specimen

**Figure 8:**
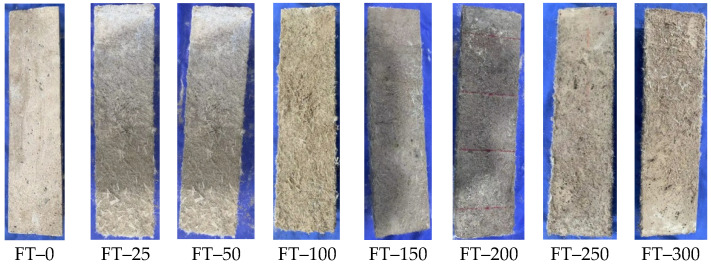
Appearance of HD–MPCC with different numbers of freeze–thaw cycles.

The appearance of the HD–MPCC after experiencing 0~300 freeze–thaw cycles is illustrated in Figure 8. As the number of freeze–thaw cycles increases, the surface of the HD–MPCC specimens becomes rougher, concurrently exposing more fibers. Despite this, due to the crack–resistant nature of fibers, no visible cracks appear on the surface of the specimens. When the specimen is placed in water, the low–temperature water will freeze. Due to the lower density of ice compared to that of water, the internal pores of the specimen will shrink as the water freezes. The subsequent thawing of the ice exerts an expansion force exceeding the surface strength of the HD–MPCC, resulting in the specimens cracking or peeling. 

#### 3.3.2. Mass Loss and Relative Dynamic Elastic Modulus

**Figure 9:**
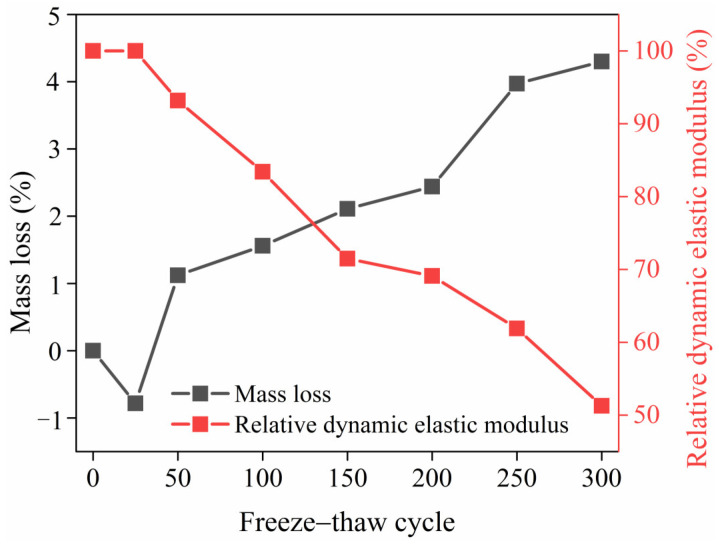
Mass loss and relative dynamic elastic modulus of HD–MPCC with different numbers of freeze–thaw cycles.

The mass loss of the HD–MPCC after experiencing 0~300 freeze–thaw cycles is shown in Figure 9. As the number of freeze–thaw cycles increases, the mass loss of the HD–MPCC decreases before subsequently increasing. During the first 25 freeze–thaw cycles, the mass of the HD–MPCC specimens increases due to the combined effect of frost expansion pressure and static water pressure. As the number of freeze–thaw cycles exceeds 25, the surface peeling of the HD–MPCC specimens becomes more severe with increasing freeze–thaw cycles, resulting in a higher mass loss.

The relative dynamic elastic modulus of the HD–MPCC following 0 to 300 freeze–thaw cycles is depicted in Figure 9. With an increasing number of freeze–thaw cycles, there is a decreasing trend in the relative dynamic elastic modulus of the HD–MPCC. Upon reaching 300 freeze–thaw cycles, the relative dynamic elastic modulus of the HD–MPCC falls below 60%, indicating that the specimens become damaged. As the freeze–thaw cycles progress, the specimens progressively peel from the surface to the interior, resulting in more severe internal damage to the HD–MPCC specimens and consequently a lower relative dynamic elastic modulus. As the number of freeze–thaw cycles surpasses 250, the internal damage to the specimens becomes more severe, causing the relative dynamic elastic modulus of the HD–MPCC to drop below 60%.

#### 3.3.3. Tensile Property of HD–MPCC after Different Numbers of Freeze–Thaw Cycles

**Figure 10:**
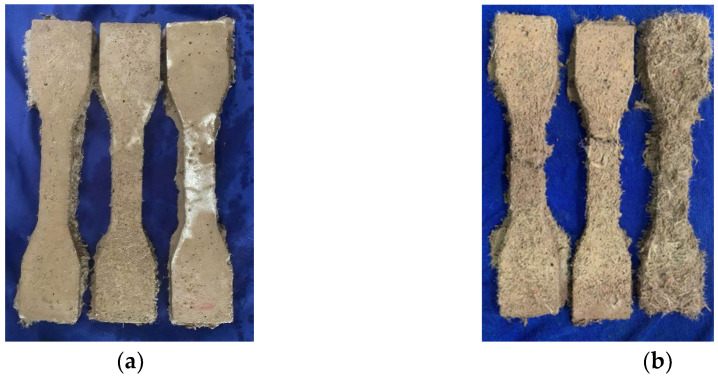
Appearance of tensile specimens for HD–MPCC with different numbers of freeze–thaw cycles: (**a**) 25 cycles and (**b**) 50 cycles.

(1) Appearance of specimens:

The appearance of the tensile specimens for the HD–MPCC after0 to 25 freeze–thaw cycles is expressed in Figure 10. After 50 freeze–thaw cycles, the surface peeling of the HD–MPCC specimens is severe, and a large amount of PVA fibers fall off, indicating specimen fracture and failure. Thus, the tensile property of the HD–MPCC was assessed after 25 freeze–thaw cycles in this study.

**Figure 11:**
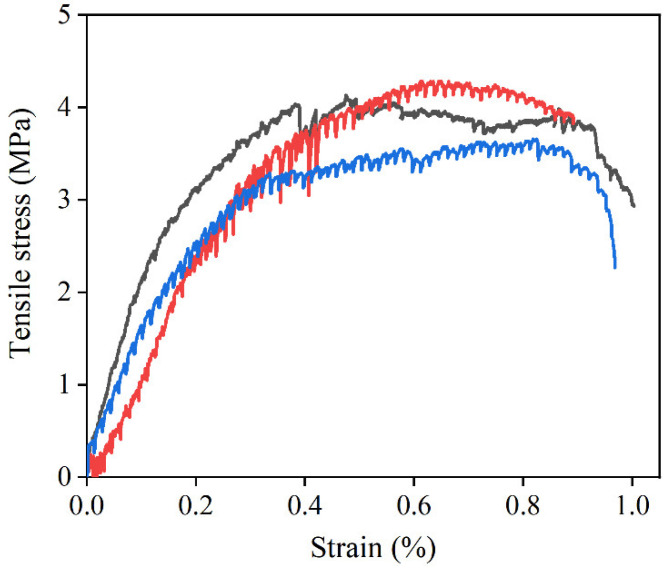
Tensile stress–strain relationship curves of HD–MPCC with different freeze–thaw cycles.

(2) Stress–strain relationship curve:

The tensile stress–strain relationship of the HD–MPCC after 25 freeze–thaw cycles is listed in Figure 11. Similar to the tensile constitutive behavior of the HD–MPCC without experiencing freeze–thaw cycles, the tensile stress–strain curve of the HD–MPCC exhibits a distinct strain hardening characteristic. This behavior is attributed to the fiber bridging effect and is independent of the freeze–thaw condition.

(3) Feature parameter:

The first–cracking strength, first-cracking strain, ultimate tensile strength, and ultimate tensile strain of the HD–MPCC are listed in Table 3. Following 25 freeze–thaw cycles, the first–cracking strain of the HD–MPCC shows no significant change, while the first–cracking strength, ultimate tensile strength, and ultimate tensile strain exhibit a decreasing trend. However, the ultimate tensile strain of the HD–MPCC remains above 0.50%, meeting the requirement for high ductility.

Compared to the specimens without freeze–thaw cycles, internal damage to the HD–MPCC occurs after 25 freeze–thaw cycles, leading to a decrease in the bearing capacity and subsequently a lower first-cracking strength. After the freeze–thaw cycles, both the matrix and fiber bridging capacity decrease, resulting in lower ultimate tensile strength and ultimate tensile strain in the HD–MPCC. 

#### 3.3.4. Flexural Property of HD–MPCC after Different Numbers of Freeze–Thaw Cycles

After undergoing 0 to 300 freeze–thaw cycles, the HD–MPCC specimens exhibit multiple cracks, and there is a main visible crack on the surface of the specimens upon ultimate failure.

**Figure 12:**
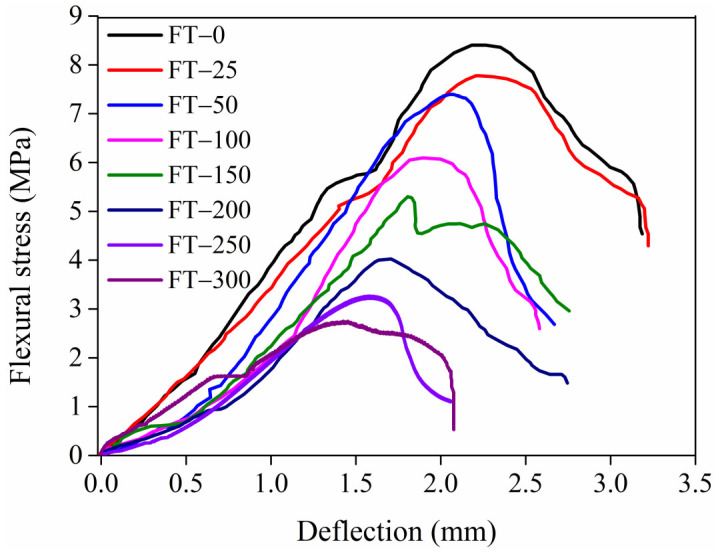
Flexural stress–deflection relationship curves of HD–MPCC with different numbers of freeze–thaw cycles.

(1) Flexural stress–deflection relationship curve:

The flexural stress–deflection relationship in the HD–MPCC after different numbers of freeze–thaw cycles is expressed in Figure 12. All the curves show four stages, namely the linear elastic stage, the nonlinear stage, the deflection hardening stage, and the deflection softening stage. 

In the early loading stages, the relationship between stress and deflection shows a linear characteristic. As the load increases, damage occurs inside the specimens. As the loads increase, the internal damages accumulate, leading to the matrix cracking. Due to the fiber bridging effect, the stress of the HD–MPCC specimens increases with increasing deflection. An obvious deflection hardening feature occurs in the flexural stress–deflection relationship curve of the HD–MPCC. 

Under the different numbers of freeze–thaw cycles, the main features in the flexural stress–deflection relationship curves of the HD–MPCC are similar. Because of the fiber bridging effect, the four stages characteristic of the curve for the HD–MPCC are independent of the freeze–thaw environment. 

(2) Feature parameter:

The flexural strength and peak deflection of the HD–MPCC decrease with increasing freeze–thaw cycles, as presented in Table 4.

The flexural strength and peak deflection of the HD–MPCC are related to the matrix, fibers, and interface properties between the fibers and the matrix of the specimens. With more freeze–thaw cycles, the internal damage to the HD–MPCC specimen matrix is severe, and fiber degradation gradually becomes more severe. In addition, the fiber bridging effect is gradually weakened, leading to a decrease in both the strength and deflection of the HD–MPCC.

### 3.4. Pore Structure

**Figure 13:**
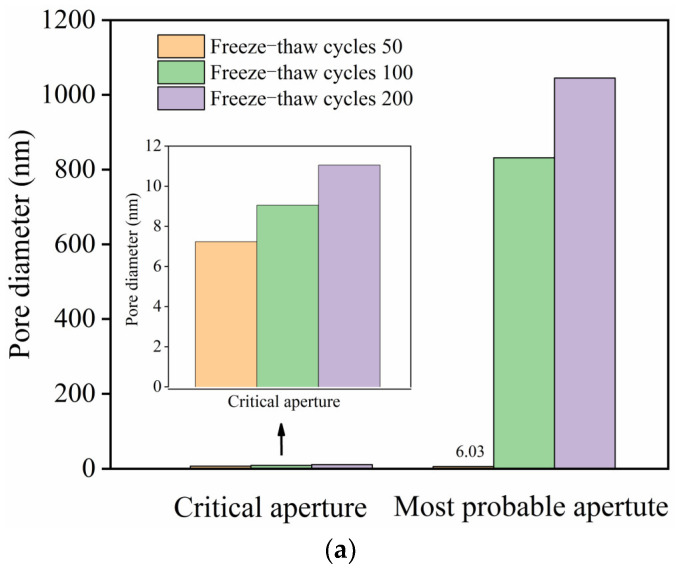

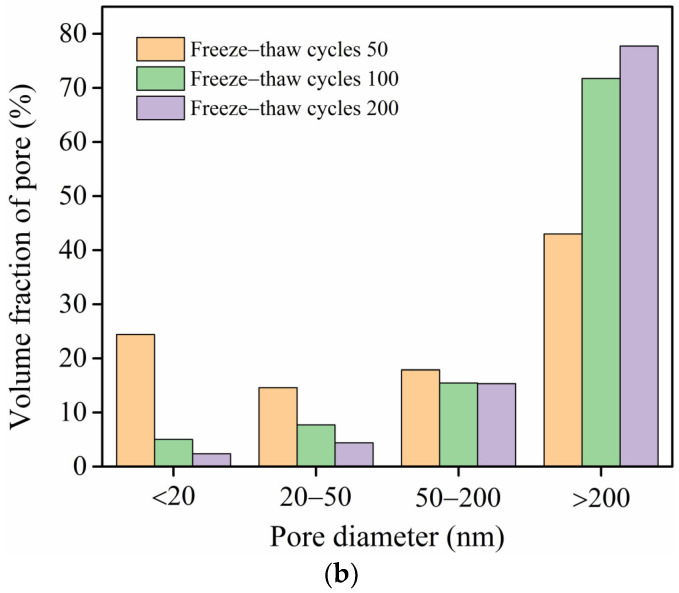
Pore distribution of the HD–MPCC with different numbers of freeze–thaw cycles. (**a**) Critical aperture and most probable aperture. (**b**) Volume fractions of different pores.

The pore structure of the HD–MPCC pure paste after undergoing 50 to 200 freeze–thaw cycles is illustrated in Figure 13. The most probable aperture and critical aperture of the pure paste show an increasing trend as the freeze–thaw cycles increase, as shown in Figure 13a.

According to the aperture division principle, the pores inside the pure paste are categorized into harmless pores (apertures smaller than 20 nm), less harmful pores (apertures ranging from 20 nm to 50 nm), harmful pores (apertures ranging from 50 nm to 200 nm), and more harmful pores (apertures larger than 200 nm). With the increasing number of freeze–thaw cycles, the volume fractions of harmless pores and less harmful pores decrease, whereas the volume fraction of more harmful pores increases, as expressed in Figure 13b.

The deterioration of the pore structure is evident with the increasing number of freeze–thaw cycles. As the most probable aperture, the critical aperture, and the more harmful pore volume fraction of the paste increase, the matrix loses its strength, resulting in reduced ultimate tensile strength and flexural strength in the HD–MPCC. Furthermore, as the freeze–thaw cycles continue, the strength of the matrix declines, and the interfacial strength between the fibers and the matrix weakens. This weakening leads to the fibers pulling out rather than fracturing. In addition, the tensile strength and tensile strain of the fibers are lower during the process of a freeze–thaw cycle, and then the fiber bridging effect becomes weaker, bringing about a reduction in the ultimate tensile strain and peak deflection of the HD–MPCC.

## 4. Conclusions

1. The expansion deformation of the HD–MPCC is observed when cured in both air and water. Within a curing period of 28 days, the increasing trend of expansion deformation is obvious, while after 28 days, the increasing trend of expansion deformation slows down. When the curing age is consistent, the expansion deformation of the HD–MPCC cured in water is greater than that of the HD–MPCC in air, indicating the poorer volume stability of the HD–MPCC in water.
2. With an increasing number of freeze–thaw cycles, the relative dynamic elastic modulus of the HD–MPC decreases, and the mass loss initially decreases then increases. The HD–MPCC specimens peel off from the surface to the inside, and the internal damage to the specimens becomes more severe with more freeze–thaw cycles.
3. After 25 freeze–thaw cycles, both the ultimate tensile strength and ultimate tensile strain of the HD–MPCC decrease. Similarly, the flexural strength and peak deflection of the HD–MPCC reduce with the increase in freeze–thaw cycles.
4. As the freeze–thaw cycles continue, the most probable aperture and critical aperture of the HD–MPCC pure paste show increasing trends, and the volume fractions of harmless pores and less harmful pores decrease, while the volume fraction of more harmful pores increases. This deterioration in pore structure results in decreased ultimate tensile strength, flexural strength, ultimate tensile strain, and flexural deflection.

## Figures and Tables

**Table 1:** Physical and mechanical properties of PVA fiber [12].

Length (mm)	Mean Diameter (μm)	Density (kg/m^3^)	Elastic Modulus (GPa)	Ultimate Tensile Strength (MPa)	Ultimate Elongation (%)
12	39	1300	30	≥1250	5~8

**Table 2:** Mix proportion of HD–MPCC (kg/m^3^).

M	P	FA	BO	RS	PS	Li	W	PVA
570	190	190	171	950	10.45	28.5	171	26

**Table 3:** Feature parameter of tensile property for HD–MPCC with 0~25 freeze–thaw cycles.

Freeze–Thaw Cycle	First–Cracking Strength (MPa)	First–Cracking Strain (%)	Ultimate Tensile Strength (MPa)	Ultimate Tensile Strain (%)
0	3.41 ± 0.53	0.03 ± 0.007	6.10 ± 0.09	1.10 ± 0.50
25	3.16 ± 0.07	0.03 ± 0.001	3.94 ± 0.18	0.82 ± 0.07

**Table 4:** Flexural strength and peak deflection of HD–MPCC with different freeze–thaw cycles.

Freeze–Thaw Cycle	Flexural Strength (MPa)	Peak Deflection (mm)
0	8.40	2.27
25	7.78	2.21
50	7.39	2.08
100	6.09	1.89
150	5.30	1.80
200	4.02	1.71
250	3.26	1.62
300	2.75	1.45

## Data Availability

The data presented in this study are available upon request from the corresponding authors.

## References

[1] Yang Z.H., Liu S.J., Wu K., Yu L., Pan F. (2023). Research progress on fiber reinforced magnesium phosphate cement composites. Mater. Rep..

[2] Zhang X.Y. (2017). Research on Development and Application Technology of Fast Repair Material MPC for Cement Pavement. Master’s Thesis.

[3] Formosa J., Lacasta A.M., Navarro A., del Valle-Zermeno R., Niubo M., Rosell J.R., Chimenos J.M. (2015). Magnesium phosphate cements formulated with a low-grade mgo by-product: Physico-mechanical and durability aspects. Constr. Build. Mater..

[4] Shi C.L., Wang X., Zhao Y.Y., Li X., Jia X.W., Qian J.S. (2024). Bond strength and interface characteristics between magnesium phosphate cement mortar and ordinary Portland cement concrete in a frigid environment. Int. J. Pavement Eng..

[5] Liu J., Yan C.W., Liu S.G., Jing L., Yin L.Q., Wang X.X. (2024). Toughness and strength of PVA-fibre reinforced magnesium phosphate cement (FRMPC) within 24 h. Constr. Build. Mater..

[6] (2018). Standard Test Method for the Mechanical Properties of Ductile Fiber Reinforced Cementitious Composites.

[7] Curosu L., Liebscher M., Alsous G., Muja E., Li H.Y., Drechsler A., Frenzel R., Synytska A., Mechtcherine V. (2020). Tailoring the crack-bridging behavior of strain-hardening cement-based composites (SHCC) by chemical surface modification of poly(vinyl alcohol) (PVA) fibers. Cem. Concr. Compos..

[8] Xu S.L., Wu P., Zhou F., Jiang X., Chen B.K., Li Q.H. (2020). A dynamic constitutive model of ultra high toughness cementitious composites. J. Zhe Jiang Univ.-Sci..

[9] Zheng Y., Zhang L.F., Xia L.P. (2018). Investigation of the behaviour of flexible and ductile ECC link slab reinforced with FRP. Constr. Build. Mater..

[10] Deng M.K., Zhang M., Ma F.D., Li F.Y., Sun H.Z. (2021). Flexural strengthening of over-reinforced concrete beams with highly ductile fiber-reinforced concrete layer. Eng. Struct..

[11] Guo L.P., Wang M., Ding C., Chai L.J. (2020). Effect of incorporating reclaimed asphalt pavement on macroscopic and microstructural properties of high ductility cementitious composites. Constr. Build. Mater..

[12] Chai L.J., Guo L.P., Chen B., Xu Y.H. (2018). Interactive effects of freeze-thaw cycle and carbonation on tensile property of ecological high ductility cementitious composites for bridge deck link slab. Constr. Build. Mater..

[13] Feng H., Liang J.H., Guo A.F., Lv L.J., Sun Z.H., Sheikh M.N., Liu F.J. (2023). Development and design of ultra-high ductile magnesium phosphate cement-based composite using fly ash and silica fume. Cem. Concr. Compos..

[14] Feng H., Nie S., Guo A.F., Lv L.J., Yu J.H. (2022). Evaluation on the performance of magnesium phosphate cement-based engineered cementitious composites (MPC-ECC) with blended fly ash/ silica fume. Constr. Build. Mater..

[15] Qiao F., Chau C.K., Li Z.J. (2010). Property evaluation of magnesium phosphate cement mortar as patch repair material. Constr. Build. Mater..

[16] Wen J.B., Tang X.S., Huang G.H., Zhu Y.R., Ma J.N. (2017). Performance testing on rapid repair materials of magnesium phosphate cement. Adv. Sci. Technol. Water Resour..

[17] Ma C., Chen B. (2016). Properties of magnesium phosphate cement containing redispersible polymer powder. Constr. Build. Mater..

[18] Li Y., Chen B. (2013). Factors that affect the properties of magnesium phosphate cement. Constr. Build. Mater..

[19] Jiang Z.W., Qian C., Chen Q. (2017). Experimental investigation on the volume stability of magnesium phosphate cement with different types of mineral admixtures. Constr. Build. Mater..

[20] Lu K.L., Wang B., Han Z.C., Ji R.J. (2022). Experimental study of magnesium ammonium phosphate cements modified by fly ash and metakaolin. J. Build. Eng..

[21] Yang Q.B., Wu X.L. (1999). Factors influencing properties of phosphate cement-based binder for rapid repair of concrete. Cem. Concr. Res..

[22] Ma H.Y., Xu B.W., Liu J., Pei H.F., Li Z.J. (2014). Effects of water content, magnesia-to-phosphate molar ratio and age on pore structure, strength and permeability of magnesium potassium phosphate cement paste. Mater. Design.

[23] Lin L.D. (2022). Study on the Stability of Magnesium Phosphate Cement Cured in Water and Its Influencing Factors. Master’s Thesis.

[24] Yang Q.B., Zhang S.Q., Wu X.L. (2002). Deicer-scaling resistance of phosphate cement-based binder for rapid repair of concrete. Cem. Concr. Res..

[25] Jia L., Nuliyanmu X.F.K.T., Chen C., Guo J., Bao D.X. (2022). Study on frost resistance of rapid-hardening magnesium phosphate-ferro-aluminate composite cement. J. Glaciol. Geocryol..

[26] Tao Q., Wang Y. (2018). Mechanical properties and frost resistance of magnesium phosphate cement concrete under negative temperature. J. Glaciol. Geocryol..

[27] Ma S.L., Cao Z., Wei C., Shao Y., Wu P.F., Zhang Z.Q., Liu X.M. (2024). Red mud-modified magnesium potassium phosphate cement used as rapid repair materials: Durability, bonding property, volume stability and environment performance optimization. Constr. Build. Mater..

[28] Wu Q.Q., Hou Y.Y., Mei J.T., Yang J.M., Gan T. (2022). Influence of synthetic limestone sand on the frost resistance of magnesium potassium phosphate cement mortar. Materials.

[29] Chai L.J., Guo L.P., Chen B., Cao Y.Z., Xu Y.H. (2019). Flexural behaviors of ecological high ductility cementitious composites subjected to interaction of freeze-thaw cycles and carbonation. J. Adv. Concr. Technol..

[30] China Building Materials Federation (2019). Magnesium Phosphate Repairing Mortar: JC/T 2537-2019.

[31] Chai L.J., Yue Z.H., Guo L.P., Chen B., Wang L.Y. (2024). Preparation and performance optimization design of high ductility magnesium phosphate cementitious rapid repair material. Acta Mater. Compos. Sinica..

[32] CCCC Highway Consultants Co., Ltd. (2015). General Specifications for Design of Highway Bridges and Culverts: JTG D60-2015.

[33] (2009). Standard for Test Method of Performance on Building Mortar.

[34] (2009). Standard for Test Methods of Long-Term Performance and Durability of Ordinary Concrete.

[35] Gao Y.X., Qin J.H., Li Z., Jia X.W., Qian J.S. (2023). Creep deformation and its effect on mechanical properties and microstructure of magnesium phosphate cement concrete. Materials.

